# The role of virtual reality on outcomes in rehabilitation of Parkinson’s disease: meta-analysis and systematic review in 1031 participants

**DOI:** 10.1007/s10072-019-04144-3

**Published:** 2019-12-06

**Authors:** Joseph Triegaardt, Thang S. Han, Charif Sada, Sapna Sharma, Pankaj Sharma

**Affiliations:** 1 Institute of Cardiovascular Research Royal Holloway, University of London, Egham, Surrey, TW20 0EX UK; 2 Department of Endocrinology, Ashford & St Peter’s NHS Foundation Trust, Chertsey, UK; 3 Department of Clinical Neuroscience, Imperial College Healthcare NHS Foundation, London, UK

**Keywords:** Cognitive function, Neurological disorders, Physical function, Quality of life

## Abstract

**Introduction:**

Parkinson’s disease (PD) is managed primarily by dopamine agonists and physiotherapy while virtual reality (VR) has emerged recently as a complementary method. The present study reviewed the effectiveness of VR in rehabilitation of patients with PD.

**Methods:**

Literature search up to June 2019 identified ten studies (*n* = 343 participants) suitable for meta-analysis and 27 studies (*n* = 688 participants) for systematic review. Standard mean difference (SMD) and 95% confidence intervals (CI) were calculated using a random effects model.

**Results:**

In meta-analysis, compared with active rehabilitation intervention, VR training led to greater improvement of stride length, SMD = 0.70 (95%CI = 0.32–1.08, *p* = 0.0003), and was as effective for gait speed, balance and co-ordination, cognitive function and mental health, quality of life and activities of daily living. Compared with passive rehabilitation intervention, VR had greater effects on balance: SMD = 1.02 (95%CI = 0.38–1.65, *p* = 0.002). Results from single randomised controlled trials showed that VR training was better than passive rehabilitation intervention for improving gait speed SMD = 1.43 (95%CI = 0.51–2.34, *p* = 0.002), stride length SMD = 1.27 (95%CI = 0.38–2.16, *p* = 0.005) and activities of daily living SMD = 0.96 (95%CI = 0.02–1.89). Systematic review showed that VR training significantly (*p* < 0.05) improved motor function, balance and co-ordination, cognitive function and mental health, and quality of life and activities of daily living.

**Conclusion:**

VR used in rehabilitation for patients with PD improves a number of outcomes and may be considered for routine use in rehabilitation.

**Electronic supplementary material:**

The online version of this article (10.1007/s10072-019-04144-3) contains supplementary material, which is available to authorized users.

## Introduction

Parkinson’s disease (PD), a progressive neurological disorder, affects the initiation and execution of voluntary movements, leading to difficulty in performing basic daily activities of living and impaired quality of life. PD is associated with shorter life expectancy [1]. Approximately 145,000 people were living with PD in the UK in 2018 and it is expected to rise to 168,000 by 2025 [2]. Dopamine agonists have been the principal drugs for treating PD since 1975 [3] but have a number of adverse effects including dopamine dysregulation syndrome, occurring in 4% of patients on long-term treatment [4], gambling addiction, excessive spending and sexual hyperfunction [4].

Physical rehabilitation is an essential complementary component to drug therapy of PD while virtual reality (VR) has increasingly been additionally applied to rehabilitation of patients with neurological conditions [5]. This technology is a computer-generated environment in which the user can perceive, feel and interact in a manner that is similar to a physical place, achieved by combining stimulation over multiple sensory channels such as sight, sound and touch [6]. Because of its ability to simulate real-world situations and cognitive and motor tasks in a safe environment, completion of VR tasks is a rewarding form of therapy for patients with PD. Other beneficial gain from VR training is the immediate feedback through the augmented reality that it provides, altering the senses that the patient experiences [7]. However, VR is a relatively novel technology and not yet routinely used in clinical practice since there are only handful of randomised controlled trials have been performed in patients with PD. The present study conducted a meta-analysis and systematic review to examine the effectiveness of VR in patients with PD.

## Methods

### Literature search

We followed guidelines from the Cochrane and PRISMA recommendations on conducting a meta-analysis [8, 9]. Literature search of MEDLINE and Google Scholar was performed up to June 2019 using the key terms: ‘virtual reality’ and ‘Parkinson’s disease’. No language or data filters were applied. The Boolean operators were used to combine search terms. Relevant studies were traced within their references.

### Selection criteria

Studies examining either idiopathic or familial PD irrespective of age, sex, drug dosage and duration of PD were included. Inclusion criteria for meta-analysis required the study to be a randomised controlled trial. Control group may be active (patients receiving an alternative therapy treatment, e.g. physiotherapy) or passive (without alternative therapy). VR may be fully immersive where the user wears a headset to view the task or non-immersive where the user views the task on computer or television monitor. The majority of studies included in meta-analysis used non-immersive Nintendo™ *Wii* (Nintendo; Redmond, WA, USA).

### Outcome measures

The outcomes for the comparative analysis were gait, stride length, balance, global motor function, activities of daily life, Parkinson’s Disease Questionnaire 39 (PDQ39) and cognitive function. It should be borne in mind that the definition of outcomes may vary between studies.

### Statistical analysis

Meta-analysis was performed using Review Manager v5.3 (Copenhagen: The Nordic Cochrane Centre, The Cochrane Collaboration, 2014). The effect size was assessed by standard mean difference (SMD) and 95% confidence intervals (CI) using random effects model. Statistical significance threshold was accepted as *p <* 0.05. Inter-study heterogeneity was assessed by I^2^ test with a value of > 50% being considered substantial heterogeneity. Risk of bias was assessed using Cochrane Collaboration’s tool [10].

## Results

**Fig. 1:**
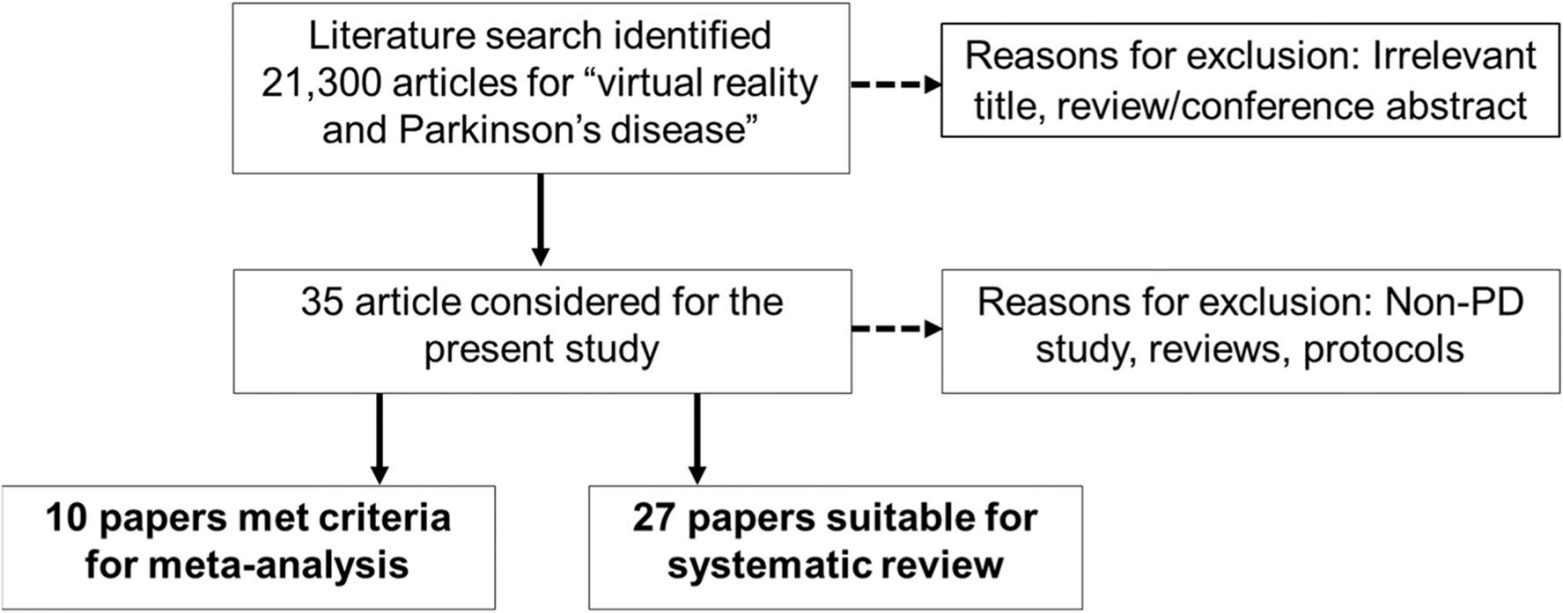
Flowchart showing process of study selection in the present study

Using the search terms ‘virtual reality’ and ‘Parkinson’s disease’ identified 21,300 articles. After screening for duplications and selection criteria, ten papers met selection criteria for meta-analysis and 27 papers suitable for systematic review (Fig. 1).

### Meta-analysis

Ten papers comprised a total of 343 participants of mean age ranging from 61.1 to 78.4 years and disease duration from 6 to 9.4 years. A variety of VR systems were used with Nintendo™ *Wii* being the most popular (in half of all studies). The remaining trials included balance boards and other commercially available systems such as GestureTek IREX videocapture system™ (Toronto, ON, Canada). Duration of the trials varied from just over 4 weeks [11] to 12 weeks [12].

Except for one study [13], all trials employed a control group that was active which included conventional physiotherapy, balance and gait training. The control for the study by Lee et al consisted of neurodevelopmental treatment followed by functional electrical stimulation [13]. Two studies included both active and passive control groups [14, 15]. Due to inter-study heterogeneity of some of the trials, not all outcomes could be used in meta-analysis.

#### Virtual reality intervention versus active intervention control

**Fig. 2:**
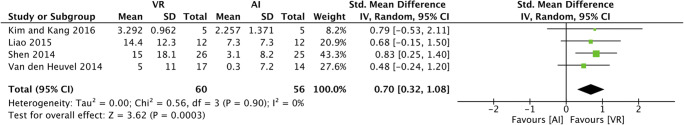
Forest plot showing effects of VR training compared with active intervention on stride length

Four trials comprising 116 patients assessed stride length [12, 15–17]. Compared with active intervention, VR training led to greater improvement of stride length: SMD = 0.70 (95%CI = 0.32 to 1.08, *p* = 0.0003). There was no evidence of inter-study heterogeneity (I^2^ = 0.0%, *p* = 0.90) (Fig. 2).

Six trials (*n* = 209 participants) assessed gait [12, 15–19]. Gait speed was similarly improved both by VR and by active intervention but there were no differences in improvement between these two methods: SMD = 0.08 (95%CI = − 0.27 to 0.44, *p* = 0.65). There was no inter-study heterogeneity (I^2^ = 34%, *p* = 0.18) (Supplementary Fig. 1).

Five trials (*n* = 166 participants) examined balance using Berg Balance scale [16–20]. Improvement in balance did not differ between VR and active intervention: SMD = 0.26 (95%CI = − 1.02 and 0.62, *p* = 0.37). There was evidence of inter-study heterogeneity (I^2^ = 61%, *p* = 0.04) (Supplementary Fig. 2).

Motor function assessed by unified Parkinson’s disease rating scale (UPDRS) was studied in three papers comprising 75 participants [11, 16, 18]. No differences in motor function were observed between VR and active intervention: SMD = − 0.38 (95%CI = − 1.45 to 0.69, *p* = 0.49). There was evidence of inter-study heterogeneity (I^2^ = 80%, *p* = 0.007) (Supplementary Fig. 3).

Quality of life was assessed in five studies comprising 176 patients [11, 15, 16, 18, 19] (four studies used PDQ39 [11, 15, 16, 18] and one used shorthand form (PDQ8) [19]). No differences in quality of life were observed between VR and active intervention: SMD = 0.20 (95%CI = − 0.16 to 0.57, *p* = 0.27). There was no inter-study heterogeneity (I^2^ = 28%, *p* = 0.23) (Supplementary Fig. 4).

#### Virtual reality intervention versus passive intervention

Two trials on balance in 44 subjects [13, 15] showed that VR was significantly better than passive intervention on balance: SMD = 1.02 (95%CI = 0.38 to 1.65, *p* = 0.002). There was no heterogeneity found between the studies (Supplementary Fig. 5).

#### Single randomised controlled trials

We found only a single study comparing VR and active intervention on activities of daily living and cognitive function comprising 32 subjects [20]. There were no differences in activities of daily living SMD = − 0.13 (95%CI = − 0.82 and 0.57, *p* = 0.72) or in cognitive function between VR and active intervention SMD = 0.08 (95%CI = − 0.61 to 0.78, *p* = 0.81).

There were also single studies comparing VR against passive intervention. The single trial of 20 subjects by Lee et al. [13] showed VR led to greater improvement in activities of daily living than passive intervention SMD = 0.96 (95%CI = 0.02 to 1.89, *p* = 0.05) and that by Liao et al [15] of 24 participants showed that VR had a greater improvement than passive intervention in gait speed SMD = 1.43 (95%CI = 0.51 and 2.34, *p* = 0.002) and in stride length SMD = 1.27 (95%CI = 0.38 to 2.16, *p* = 0.005).

#### Risk of bias of randomised controlled trials

**Fig. 3:**
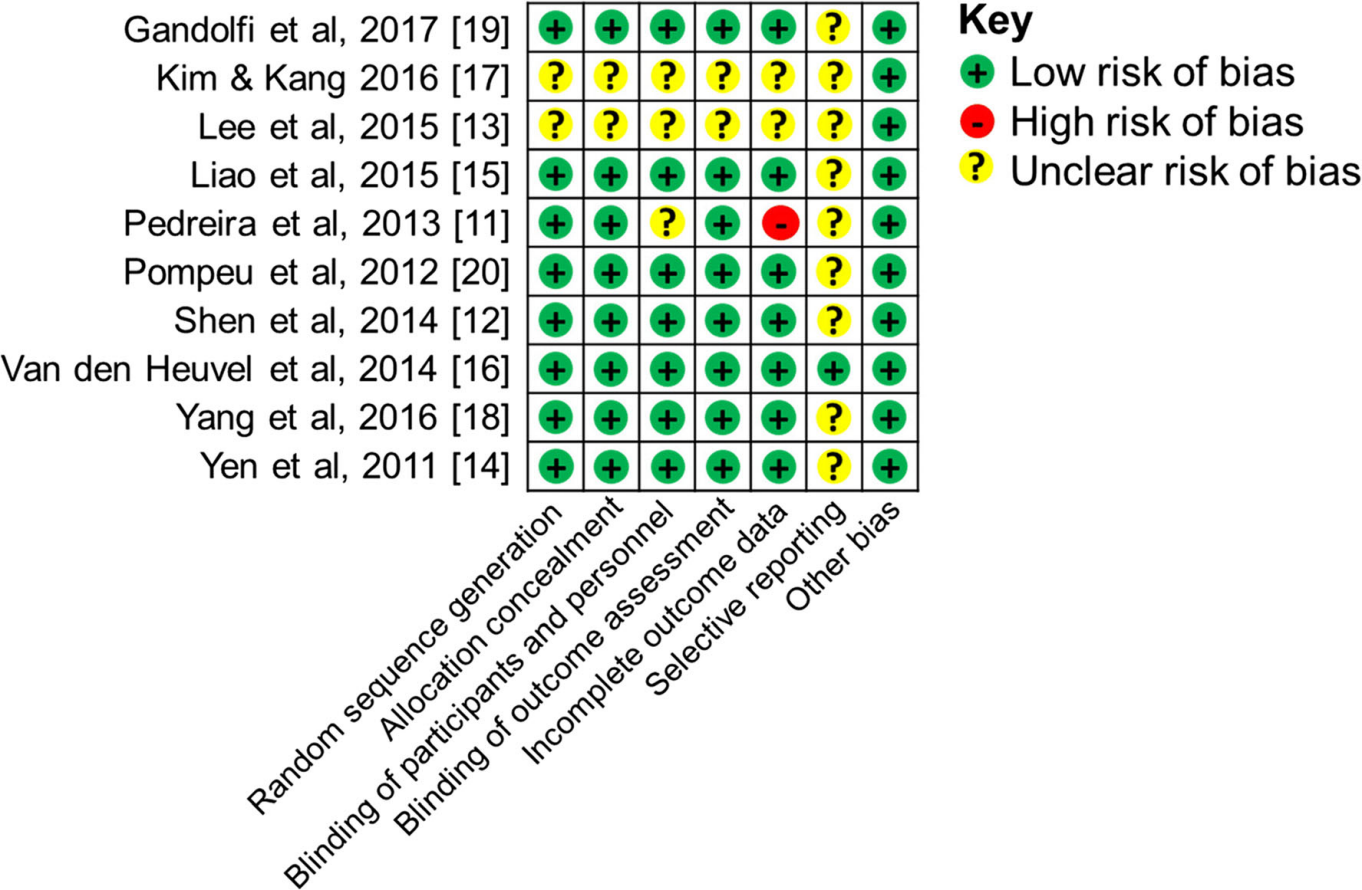
Risk of bias for meta-analysis studies

Figure 3 shows risk of bias assessments for the ten randomised controlled trials in the present study. *Random sequence generation and allocation* was examined and found only 2 papers [13, 17] to have an unclear risk of bias. The remaining trials reported methods of randomisation, therefore were considered to have low risk of bias. All the trials were blinded apart from two [13, 17]. The trial by Pedreira et al. had an unclear risk of bias while the remaining trials, which were blinded, had low risk of bias [11]. Three studies had an unclear risk of incomplete outcome data bias [13, 15, 17]. The rest of the trials either had no drop-outs or they used intent to treat analysis to compensate for drop-outs [12, 14, 16, 18, 19] while the study by Pedreira et al. had a high risk of bias [11]. *Selective reporting bias* was unclear in most studies as protocol was not mentioned, only 1 paper reported the study protocol [16], therefore was considered to have a low risk of bias.

### Systematic review

A total of 27 papers comprising 688 participants met selection criteria for the systematic review. Participant characteristics and study features are shown in Supplementary Table 1. The mean age ranged between 60.3 and 72 years and PD duration between 5.1 and 9.8 years. The majority papers examined the effect of VR on motor outcomes including balance, gait or activities of daily living while quality of life was assessed in 23 papers. Significant improvements in at least one of the outcomes by VR were reported in 18 papers. Nintendo™ *Wii* was the most popular VR system (11 studies) followed by Xbox Kinect™ (Microsoft Corporation, Redmond, WA, USA) (four studies) while two studies used custom made VR systems. The remaining ten studies used other commercially available VR systems such as Oculus Rift head-mounted displays (Facebook Technologies, LLC, Menlo Park, CA, USA).

Studies often assessed the effects of VR on different outcomes. For clarity, outcomes were classified into groups of (1) *motor function* which includes gait and stride length, endurance, walking distance, Timed Up and Go, Sitting to Standing Test and tapping; (2) *balance and co-ordination*; (3) *cognitive function and mental health* including Geriatric Depression Scale and *(*4) *quality of life and activities of daily living*.

#### Motor function

VR training has been shown to improve Timed Up and Go a decrease of 1.9 s in Timed Up and Go test compared with a decrease of 1.2 s in control group (*p* = 0.040) [21]. Zettergren et al. found an improvement in Timed Up and Go by 34% as well as gait speed by 42% after VR training twice a week for 8 weeks [22]. Badarny et al. observed that 65% of patients improved either gait speed or stride length or both by more than 10% with VR training (*p* = 0.002) [23]. These findings are supported by a study by Palacios-Navarro et al. who observed that VR training significantly increased in gait speed (*p* = 0.002), reducing the completion time from 12 to 10 s [24]. de Melo et al. found that compared with conventional training, VR intervention led to faster gait speed (*p* = 0.031) and higher Borg score (a measure of physical fitness) (*p* = 0.005) [25], and similarly, Mirelman et al. showed gait speed increased in normal walking (*p* = 0.006) and in obstacle negotiation (0.001) and stride increased in normal walking (*p* = 0.043) and in obstacle negotiation (*p* = 0.019) [26]. Mirelman et al. also found that UPDRS was significantly (*p* = 0.020) increased with VR training [26]. VR training has also led to improvements in Sitting to Standing Test (*p* = 0.010), 10-Metre Walk Test and Performance Orientated Mobility Assessment Test (*p* = 0.050) [21] and 6-Minute Walk Test (*p* = 0.043) [17]. There was only one study on tapping frequency showing VR training had no significant effects in patients with PD [27]. A number of other studies found VR training led to improvement in gait speed [12, 15, 18, 20], stride length [15], Timed Up and Go [15, 18, 28] and UPDRS [20, 28]. Although Yang et al. did not find significant improvement in UPDRS [18].

#### Balance and co-ordination

Esculier et al. found that VR training in PD patients led to significant improvement in one leg stance duration (*p* = 0.020), community balance and mobility score by 15 points compared with improvement in control group of 7.5 points (*p* = 0.001) [21]. VR training improved activities-specific balance confidence (ABC) test by 6% (*p* = 0.025) [12] but not balance assessed by Centre of Pressure Length score [29]. In contrast, Loureiro et al. showed VR training improved Borg scale (*p* = 0.046), Berg Balance Scale (*p* = 0.046) and functional reach to the left (*p* = 0.043) and right (*p* = 0.028) [30].

Balance has been shown to improve by VR training in a number of other studies [13, 18, 20, 31]. VR training also improved unipedal stance test (*p* = 0.050) [20] and Balance Evaluation System Test score from 74.1 to 88.9 [32]. Severiano et al. found improvement in the Dizziness Handicap Inventory (*p* = 0.022) after VR intervention [31]. Gandolfi et al. investigated the effect of VR and Sensory Balance Integration Training on balance showing both methods led to significant (*p* = 0.040) improvement of Berg Balance Scale scores: VR group improved by 3.74 and Sensory Balance Integration Training group by 4.21 points. Although there were no differences in satisfaction rates between study groups, the cost of VR rehabilitation was cheaper than that of Sensory Balance Integration Training group by €5600 [19].

Herz et al. found VR training led to significant increases in Purdue Pegboard Test score (*p* = 0.011), Timed Tap score (*p* = 0.003) and 9 hole peg test (*p* = 0.033) [28], while Ma et al. observed that VR training resulted in more force (*p* = 0.005) and quicker time (*p* = 0.005) to reach for stationery balls than the control [33]. A study by van den Heuvel et al. found VR training did not improve Functional Reach Test [16]. A study by Kim and Kang showed VR training resulted in significant greater increase than control in weight bearing distribution difference (with eyes open) (*p* = 0.038) and Berg Balance Scale (*p* = 0.043), improvement in mediolateral and anteroposterior sway length, ground reaction force and step length (*p* = 0.043) [17], while Liao et al. found improvement in Fall Efficacy Scale (*p* < 0.001) [15] and Yen et al. found improvement in sensory integration for postural control (*p* < 0.001) [14].

#### Cognitive function and mental health

Pompeu et al. showed VR training significant improved Montreal cognitive assessment (*p* = 0.001) [20]. Cipresso et al. assessed Executive Function after VR training found that patients with PD with normal cognition were better than those with PD with mild cognitive impairment in performing Clock Drawing Test (*p* = 0.001), Phonological Fluency Test (*p* = 0.001), semantic verbal fluency test (*p* = 0.001) and the Tower of London Test (*p* = 0.001) as well as strategies (*p* = 0.001) [34]. There were no group differences in performing Virtual Multiple Errands Test or rule breaks. Mirelman et al. found VR training did not improve mistakes during Serial Subtraction Test (*p* = 0.160) [26].

Lee et al. found VR training led to a significant decrease in Beck Depression Inventory score (*p* < 0.050) [13] while Herz et al. found no improvement on Hamilton Depression Scale scores [28].

#### Quality of life and activities of daily living

VR training has been shown to significantly improve quality of life by several studies using PDQ39 [11, 15, 18, 20, 28] while Severiano et al. found significant improvement in quality of life assessed by Short Form-36 by tight rope (*p* = 0.045) and ski slalom games (*p* = 0.012) [31]. VR training also led to improvement in activities of daily living assessed by Modified Barthel Index (*p* < 0.050) [13] or by Nottingham Extended Activities of Daily Living Test score (*p* = 0.015) [28].

#### Risk of bias in systematic review studies

**Fig. 4:**
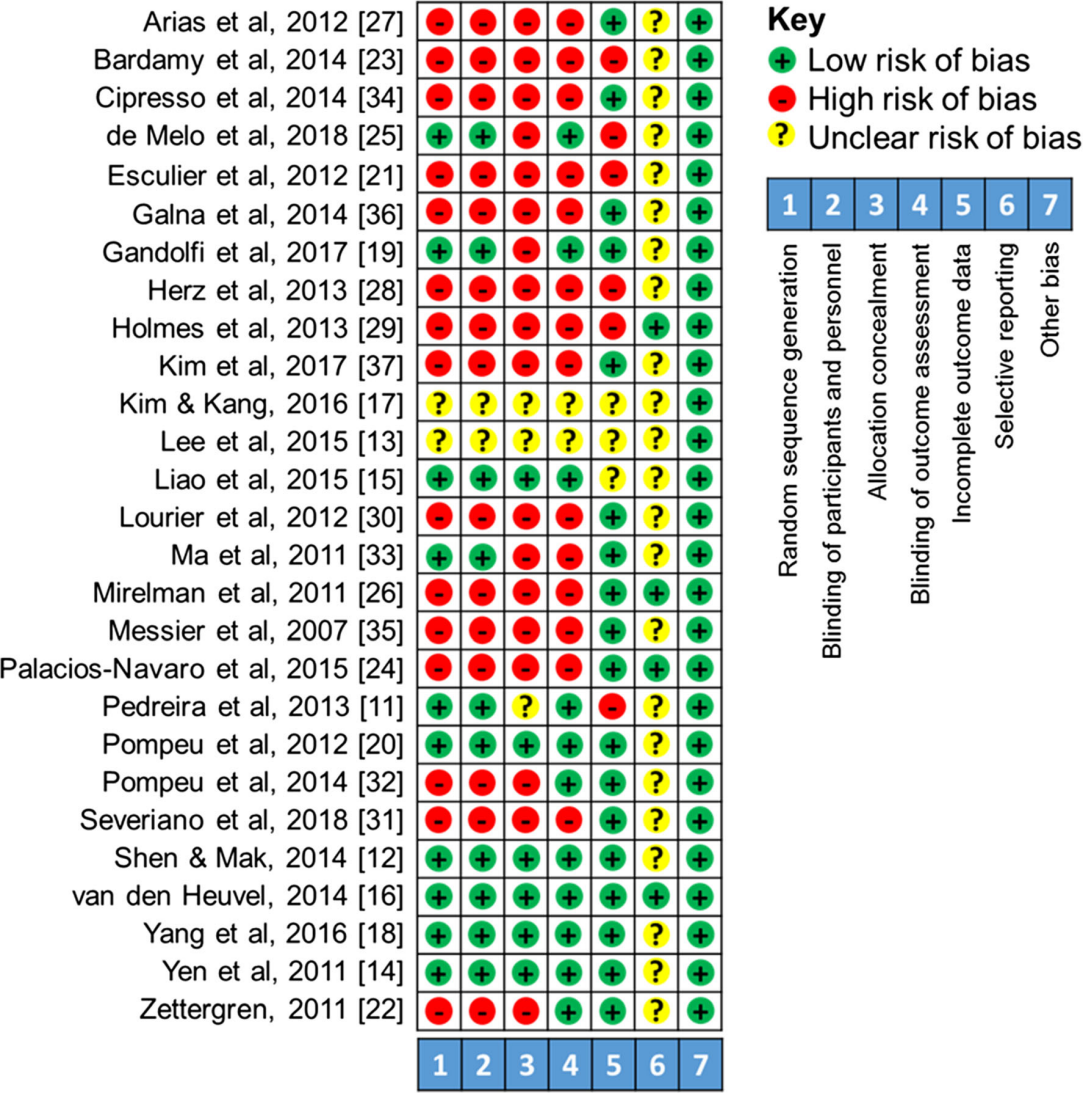
Risk of bias for systemic review studies

Figure 4 shows risk of bias for systematic review. For *random sequence generation and* allocation, 15 studies showed a high risk, two unknown risk, ten low risk of allocation bias. *Blinding* was split into blinding of participants and of outcome assessment; 18 studies showed a high risk, three unclear and six low risk of bias. Blinding of the examiners is arguably easier in studies that require high level of patient interaction; thus, more studies were found to have a low risk of bias for blinding of the examiners; 14 studies showed a high risk, 11 low risk and two unclear risk of bias. *Incomplete outcome data* examined the number of drop-outs and found six studies to have a high risk, three unclear risks and 18 low risk of bias for incomplete data. *Selective reporting bias* was based on the availability of study protocol; four studies had a low risk while 23 had an unknown bias.

## Discussion

The present meta-analysis and systematic review of over a thousand participants found VR training improved a number of outcomes in patients with PD including motor functioning, balance and co-ordination, cognitive function and quality of life. VR is a relatively new technology applied to the field of clinical medical; therefore, there is a paucity of data on its effectiveness. Findings from our study of a relatively large number of subjects are valuable in providing scientific evidence to healthcare specialists when considering to integrate VR training into rehabilitation programme.

There are a number of benefits in using VR with or without conventional physiotherapy. The use of commercially available sources of VR has the potential to allow an increased number of patients to access care from the comfort of their own home, reducing the need to travel to access care. VR training is safe and interesting [7]. The lower cost is also attractive to healthcare organisations. A recent study has found that the cost of VR rehabilitation for 36 patients with PD over 7 weeks was €23,299 compared to €28,890 for 34 patients using balance training [19]. This saving of €5,590 is enormous if most of patients with PD undergo VR training.

Pompeu et al. [32] have demonstrated that patients’ VR game performance increased over time (from first to last session) (*p* < 0.05) and found no adverse effects from playing the games. There were few studies showing poorer outcomes after VR training including that of Messier et al. who found patients with PD were worse at visuomotor learning compared to the age matched controls [35]. Galna et al. assessed game design and game feasibility found that patients preferred the games to be at a slow pace over narrative driven games. Patients tended to enjoy games with no negative effects. Most participants felt safe using the equipment and would prepare to use the system at home. Some patients had difficulty with stepping tasks and some of the objects within the game [36]. Kim et al. observed no significant adverse effects such as sickness or balance issues as a result of exposure to VR while levels of stress decreased and arousal increased [37].

The value of VR in rehabilitation of patients with neurological disorders has been recognised by a number of clinical specialists for about two decades [38–40]. The use of VR rehabilitation for other neurological conditions such as stroke has been shown to have similar beneficial effects to those used in patients with PD including physical or motor function [41–43], activities of daily living [42] and quality of life [43].

The exact mechanisms of how VR improves outcomes measure are not yet fully understood. VR has interested neuroscientists in the field of neuroergonomics research as a tool for neurorehabilitation [44] because of the human brain plasticity which enables it to adapt to environmental pressure, experiences and challenges, which could be replicated by VR, including patients with brain disorders including patients with PD or stroke [45–47].

### Limitations

The major limitation lies in its small number of studies and participants, which is expected for a novel technology. We have therefore assessed comprehensively the risk of bias to address limitations of each individual paper. Most of the studies examined the effects of VR over a relatively short period; therefore, long-term outcomes are not known. The variety of different VR systems (non-immersive and fully immersive) used by different trials may have affected the results since some system may confer greater advantage than others. VR technology is still evolving and higher quality and unified system may be available for specific use in rehabilitation of patients with different needs.

In conclusion, VR used in rehabilitation for patients with PD improves a number of outcomes and may be considered for routine use in rehabilitation.

## Electronic supplementary material

ESM 1 (PDF 500 kb)
