# Supplementary material for: The role of virtual reality on outcomes in rehabilitation of Parkinson’s disease: meta-analysis and systematic review in 1031 participants
Source: Neurol Sci. 2019 Dec 6;41(3):529–36. doi: 10.1007/s10072-019-04144-3 (PMC7040061; doi:10.1007/s10072-019-04144-3)
Supplement: Supplementary file 1 — (PDF 500 kb) [file 10072_2019_4144_MOESM1_ESM.pdf]

Supplementary Fig. 1 Forest plot of six studies comparing the effects of VR training and active intervention on gait speed.

	VR			AI				Std. Mean Difference
Study or Subgroup	Mean	SD	Total	Mean	SD	Total	Weight	IV, Random, 95% CI
Gandolfi 2017	3	24.2153	36	14	24.2153	34	26.6%	−0.45 [−0.92, 0.03]
Kim and Kang 2016	3.292	0.962	5	2.257	1.371	5	6.4%	0.79 [−0.53, 2.11]
Liao 2015	11.9	16.2	12	8.3	9	12	14.1%	0.27 [−0.54, 1.07]
Shen 2014	9.6	21	26	10.2	11.1	25	22.9%	−0.03 [−0.58, 0.51]
Van den Heuvel 2014	14.5	35.7	17	−0.1	17.4	14	16.5%	0.49 [−0.23, 1.21]
Yang 2016	4.09	2.98	11	3.17	2.86	12	13.6%	0.30 [−0.52, 1.13]
**Total (95% CI)**			**107**			**102**	**100.0%**	**0.08 [−0.27, 0.44]**

Heterogeneity: $Tau^2 = 0.06$; $Chi^2 = 7.56$, df = 5 (P = 0.18); $I^2 = 34\%$

Test for overall effect: Z = 0.45 (P = 0.65)

Std. Mean Difference
IV, Random, 95% CI

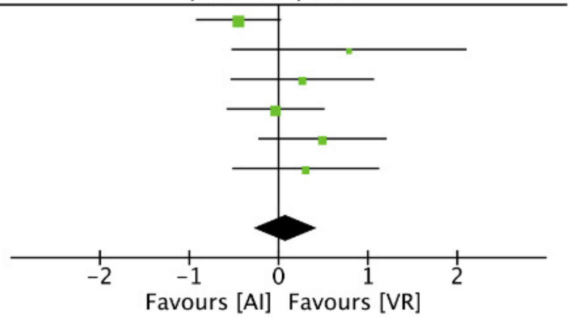

Supplementary Fig. 2 Forest plot of five studies comparing the effects of VR training and active intervention on balance.

	VR			AI				Std. Mean Difference
Study or Subgroup	Mean	SD	Total	Mean	SD	Total	Weight	IV, Random, 95% CI
Gandolfi 2017	3.74	6.4218	36	4.21	6.4218	34	28.7%	−0.07 [−0.54, 0.40]
Kim and Kang 2016	6.8	1.48	5	2	1	5	5.1%	3.43 [1.14, 5.73]
Pompeu 2012	1.4	2.6	16	1.1	2.1	16	23.2%	0.12 [−0.57, 0.82]
Van den Heuvel 2014	0.82	1.67	17	−0.21	2.33	14	22.6%	0.50 [−0.22, 1.22]
Yang 2016	3.36	2.38	11	4.17	5.01	12	20.3%	−0.20 [−1.02, 0.62]
**Total (95% CI)**			**85**			**81**	**100.0%**	**0.26 [−0.30, 0.82]**

Heterogeneity: $Tau^2 = 0.23$; $Chi^2 = 10.30$, df = 4 (P = 0.04); $I^2 = 61\%$

Test for overall effect: Z = 0.90 (P = 0.37)

Std. Mean Difference
IV, Random, 95% CI

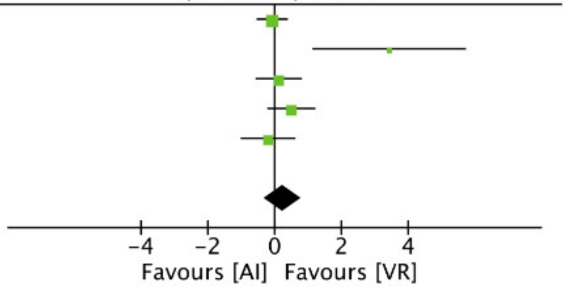

Supplementary Fig. 3 Forest plot of three studies comparing the effects of VR training and active intervention on motor function.

	VR			AI				Std. Mean Difference
**Study or Subgroup**	**Mean**	**SD**	**Total**	**Mean**	**SD**	**Total**	**Weight**	**IV, Random, 95% CI**
Pedreira 2013	−9.14	12.43	11	1.88	9.37	11	32.4%	−0.96 [−1.86, −0.07]
Van den Heuvel 2014	0.53	6.27	17	5.5	3.9	13	34.5%	−0.90 [−1.66, −0.14]
Yang 2016	2.55	5.96	11	−3.17	8.73	12	33.1%	0.73 [−0.12, 1.58]
**Total (95% CI)**			**39**			**36**	**100.0%**	**−0.38 [−1.45, 0.69]**

Heterogeneity: $Tau^2 = 0.72$; $Chi^2 = 9.97$, df = 2 (P = 0.007); $I^2 = 80\%$
Test for overall effect: Z = 0.69 (P = 0.49)

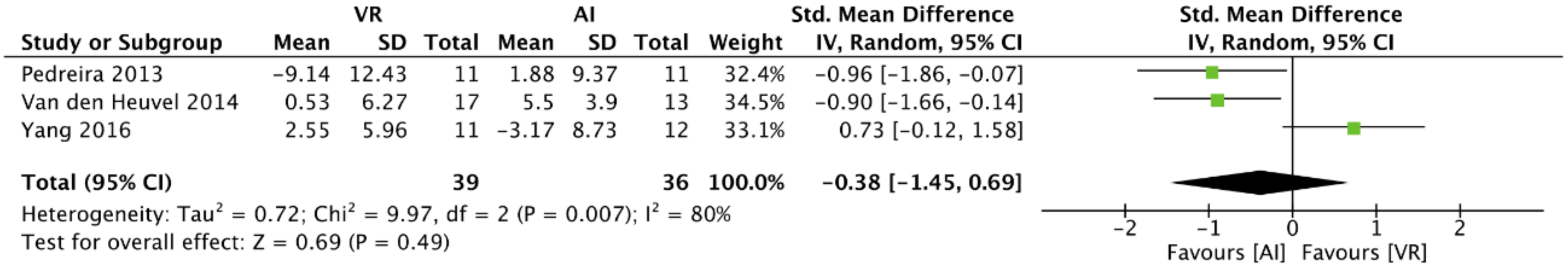

Supplementary Fig. 4 Forest plot of five studies comparing the effects of VR training and active intervention on quality of life.

	VR			AI				Std. Mean Difference
**Study or Subgroup**	**Mean**	**SD**	**Total**	**Mean**	**SD**	**Total**	**Weight**	**IV, Random, 95% CI**
Gandolfi 2017	−6.56	14.5505	36	−6.32	14.5505	34	32.7%	−0.02 [−0.49, 0.45]
Liao 2015	15.7	18.2	12	11.4	8.2	12	16.0%	0.29 [−0.51, 1.10]
Pedreira 2013	9.14	12.43	16	−1.88	9.37	15	17.8%	0.97 [0.22, 1.72]
Van den Heuvel 2014	0	12.4	16	0.63	5.7	12	17.9%	−0.06 [−0.81, 0.69]
Yang 2016	5.34	11.96	11	5.27	11.96	12	15.6%	0.01 [−0.81, 0.82]
**Total (95% CI)**			**91**			**85**	**100.0%**	**0.20 [−0.16, 0.57]**

Heterogeneity: $Tau^2 = 0.05$; $Chi^2 = 5.58$, df = 4 (P = 0.23); $I^2 = 28\%$
Test for overall effect: Z = 1.09 (P = 0.27)

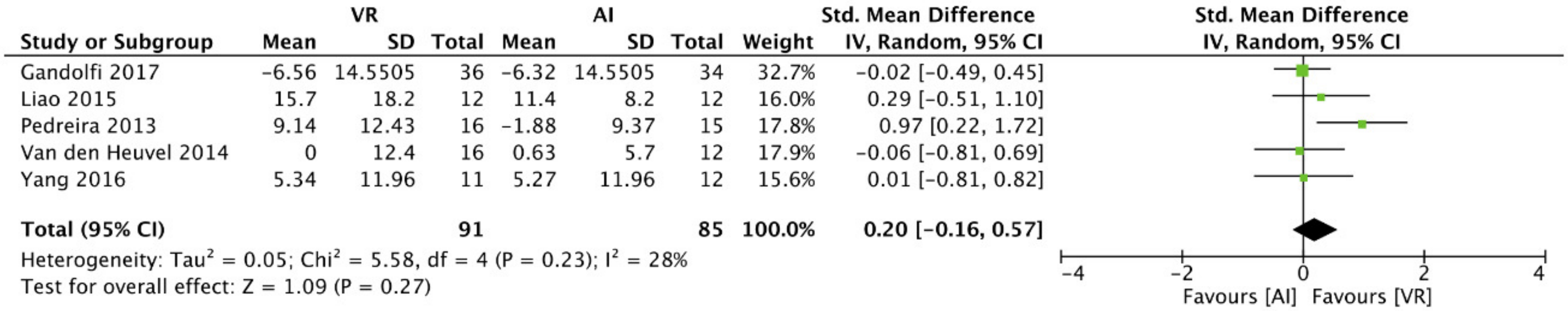

Supplementary Fig. 5 Forest plot of five studies comparing the effects of VR training and passive intervention on balance.

	VR			Passive Intervention				Std. Mean Difference
**Study or Subgroup**	**Mean**	**SD**	**Total**	**Mean**	**SD**	**Total**	**Weight**	**IV, Random, 95% CI**
Lee 2015	2.1	2.3	10	0.4	0.8	10	46.2%	0.95 [0.01, 1.88]
Liao 2015	2.9	2.2	12	0.7	1.7	12	53.8%	1.08 [0.21, 1.95]
**Total (95% CI)**			**22**			**22**	**100.0%**	**1.02 [0.38, 1.65]**

Heterogeneity: $Tau^2 = 0.00$; $Chi^2 = 0.04$, df = 1 (P = 0.84); $I^2 = 0\%$
Test for overall effect: Z = 3.14 (P = 0.002)

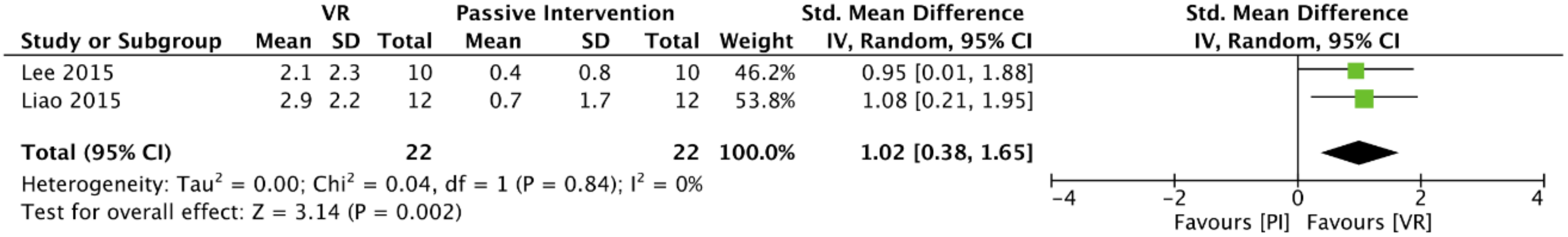

Supplementary Table 1. Characteristics of systematic review studies.

Studies	n	Mean ±SD age (yrs)	Mean ±SD PD duration (yrs)	Hoehn Yahr scale	PD drugs	M: F	Source of VR	Primary and secondary outcomes
Arias et al (2012)	PD (n=10)	69.9 ±11.2	N/A	N/A	N/A	5: 5	HMD, Vuzix iWear VR920 glasses and motion tracker.	Detect differences in tapping frequency between groups, check the reliability of using VR.
	Young control (n=12)	24.3 ±4.9				7: 5		
	Older control (n=12)	66.6 ±10.1				5: 7		
Badarny et al (2014)	VR (n=20)	71.25	5.28	2.5-4	Y	N/A	Belt-mounted unit, housing motion sensors and digital processing components, and delivered by a micro-display.	Gait speed and stride length.
Cipresso et al (2014)	PD-NC (n=15)	69.0 ±8.1	N/A	N/A	N/A	9: 6	NeuroVR.	Investigate the differences in executive function (EF) and VMET scores between groups.
	PD-MCI (n=15)	68.1 ±9.4				8: 7		
	Control (n=15)	61.7 ±5.2				6: 9		
de Melo et al (2018)	VR=12	60.3 ±9.3	N/A	1.4 ±0.5	Y	11: 1	Xbox Kinect.	Primary: 6MWT (gait speed and endurance) Secondary: physical fitness (SpO_2, Heart rate, blood pressure, BORG).
	Treadmill=13	61.0 ±10.7	N/A	1.5 ±0.7	Y	12: 1		
	Conventional training=12	65.6 ±13.0	N/A	2.1 ±0.9	Y	5: 7		
Tremblay et al (2012)	PD=11	61.9 ±11.0	8.5 ±3.6	N/A	Y	6: 5	Wii Fit.	Primary: evaluate the effect of VR on balance and functional ability of PD patients. Secondary: compare results with healthy people.
	Control=9	63.5 ±12.0				5: 4		
Galna et al. 2014	Game design: 2	N/A	N/A	N/A	N/A	N/A	Xbox Kinect.	Primary: Assess game design Secondary: assess game feasibility.
	Feasibility: 9	68.2 ±8.3	N/A	1.8 ±0.7	Y	3: 6		
Gandolfi et al. 2017	VR=36	67.5 ±7.2	6.2 ±3.8	2.5	Y		Tele-Wii protocol, remote physiotherapist, Wii + balance board, laptop with skype.	Primary: BBS. Secondary: DGI, 10MWT, MCID, PDQ8, satisfaction of patients and comparison of costs between groups.

	SIBT=34	69.8 ±9.4	7.5 ±3.9	2.5	Y			
Herz et al. 2013	VR=20	66.7 ±7.2	5.5 ±4.3	2	Y	13: 7	Nintendo Wii.	Primary: change in NEADL<br>Secondary: quality of life and motor function: changes in the UPDRS, the 9-hole peg test, the Purdue Pegboard Test, a timed tapping task, TUG, HAMD, and the PDQ-39.
Holmes et al. 2013	VR=11	66.6 ±5.9	8.1±3.8	2.3 ±0.4	Y	7: 4	Nintendo Wii.	Balance Centre of Pressure Length (COPL)
Kim et al, 2017	PD-VR=11	65 ±7	N/A	N/A	Y	3: 8	Oculus Rift DK2.	Primary: measure adverse effects of using VR.<br>Secondary: measure levels of arousal.
	Elderly control=11	66 ±3				3: 8		
	Young control=11	28 ±7				5: 6		
Kim & Kang 2016	VR=5	76.2 ±3.9	N/A	2.2 ±0.4	N/A	2: 3	IREX.	Primary: Balance and gait speed<br>Secondary: falls efficacy.
	Control=5	78.4 ±5.8	N/A	2.4 ±0.5	N/A	3: 2		
Lee et al. 2015	VR=10	68.4 ±2.9	N/A	N/A	N/A	5: 5	Nintendo Wii.	Primary: the effect of VR on balance (BBS).<br>Secondary: the effect of VR on activities of daily living and depression (MBI and BDI).
	Control=10	70.1 ±3.3	N/A	N/A	N/A	5: 5		
Liao et al, 2015	VR=12	67.3 ±7.1	7.9 ±2.7	2.0 ±0.7	Y	6: 6	Nintendo Wii Fit.	Primary: gait speed, stride length, obstacle clearance and dynamic balance.<br>Secondary: Sensory organisation test (SOT), timed up and go test,<br>Falls efficacy and PDQ39.
	Traditional exercise=12	65.1 ±6.7	6.9 ±2.8	2.0 ±0.8	Y	6: 6		
	Control=12	64.6 ±8.6	6.4 ±3.0	1.9 ±0.8	Y	5: 7		
Loureiro et al, 2012	VR=6	65 ±13	N/A	2-3	N	N/A	Nintendo Wii Fit.	Primary: Motor skills (Borg scale, BBS, TUG, functional reach tests) Secondary: quality of life (Nottingham scale).
Ma et al, 2011	VR=17	64.8 ±8.5	5.3 ±4.4	2.06 ±0.24	Y	8: 9	OpenGL.	Reaching with favoured hand to grab.<br>real stationery and moving balls down a ramp
	Control=16	68.1 ±7.4	5.2 ±3.4	2.2 ±0.4	Y	10: 6		
Mirelman et al, 2011	VR=20	67.1 ± 6.5	9.8 ±5.6	2.2 ±0.4	Y	14: 6	LED lights on shoes, treadmill and screen.	Primary: gait speed,<br>endurance testing, stride length, obstacle negotiation.<br>Secondary: cognitive and clinical measures (UPDRS and PDQ39)

Messier et al. 2007	PD=8	71.1 ±5.5	10.25 ±3.06	2.6 ±0.2	N	6: 2	SGI Octane.	Accuracy in horizontal and vertical dimension
	Healthy young=10	27	N/A	N/A	Y			
	Healthy elderly=10	68.5	N/A	N/A	Y			
Palacios-Navaro et al, 2015	VR=7	66.8 ±3.5	N/A	N/A	Y		Xbox Kinect.	10MWT
Pedreira et al, 2013	VR=22	61.1 ± 8.2	8.6 ±4.6	2.5 ±0.6	N/A	15: 7	Nintendo Wii.	Quality of life (PDQ39)
	physiotherapy=22	66.2 ±8.5	7.3 ±6.6	2.5 ±0.6	N/A	16: 6		
Pompeu et al. 2012	VR=16	60-85	N/A	N/A	Y	N/A	Nintendo Wii.	Primary: independent performance of daily tasks (UPDRS II).<br>Secondary: Dynamic balance (BBS), static balance (unipedal stance test), cognitive performance (Montreal cognitive assessment).
Pompeu et al, 2014	VR=7	72 ±9	N/A	2.1 ±0.6	Y	6: 1	Xbox Kinect.	Primary: feasibility and safety of using VR (game scores and adverse events).<br>Secondary: clinical outcomes (BESTest, DGI, 6MWT and PDQ39).
Severiano et al. 2018	VR=16	58.7 ±18.7	5.1 ±3.2	N/A	N/A	N/A	Nintendo Wii.	Primary: dizziness handicap index (DHI), BBS and SF36 after 20 VR sessions.<br>Secondary: improvement of scores in each game after 20 VR sessions.
Shen & Mak 2014	VR=22	63.3 ±8.0	8.1 ±4.3	2.4 ±0.5	Y	13: 9	Computerised dancing system + SMART EquiTest balance master.	Primary: balance confidence (ABC).<br>Secondary: balance and gait performance (LOS and SLS.
	Physiotherapy=23	65.3 ±8.5	6.6 ±4.0	2.5 ±0.5	Y	12: 11		
van den Heuvel et al. 2014	VR=17	66.3 ±6.4	9.0 (range: 4.0-13.3)	2.5 (range: 2-3)	Y	12: 5	commercially available interactive dynamic balance exercises (Motek Med- ical, Amsterdam, the Netherlands).	Primary: FRT.<br>Secondary: balance and gait (BBS, SLS, 10MWT). Health status and participation (UPDRS, FES, PDQ39, HADS, MFI).
	Physiotherapy=16	68.8 ±9.7	8.8 (range: 2.5-11.5)	2.5 (range: 2-3)	Y	8: 8		
Yang et al, 2016	VR=11	72.5 ±8.4	9.4 ±3.6	3 (range: N/A)	Y	4: 7	VR balance board.	Primary: BBS.<br>Secondary: DGI, TUG, PDQ39, UPDRS.
	Control=12	75.4 ±6.3	8.3 ±4.1	3 (range: N/A)	Y	5:7		
Yen et al. 2011	VR=14	70.4 ±6.5	6.0 ±2.9	2.6 ±0.5	Y	12:2	VR balance board.	SOT, auditory arithmetic subtraction task.

	Physiotherapy=14	70.1 ±6.9	6.1 ±3.3	2.4 ±0.5	Y	12:2		
	Control=14	71.6 ±5.8	7.8 ±4.2	2.6 ±0.4	Y	9:5		
(Zettergren et al. 2011)	VR=1	69	N/A	N/A	N/A	1	Nintendo Wii Fit.	Primary: measure effect of VR on: gait, TUG, BBS and GDS.<br>Secondary: measure scores of VR games.

Abbreviations: HMD; Head mounted display. VR; virtual reality. PD-NC; Parkinson's disease normal cognition. PD-MCI; Parkinson's disease mild cognitive impairment. VMET; Virtual multiple errands test. 6MWT; 6-minute walk test. SpO_2; peripheral oxygen saturation. CBM; community balance and mobility assessment. TUG; timed up and go test. ABC; activities balance and confidence scale. STST; sitting to standing test. POMA; performance orientated mobility assessment. SIBT; sensory balance integration training. BBS; Berg balance scale. DGI; Dynamic gate index. 10MWT; 10-meter walk test. MCID; minimal clinically important difference. PDQ8; Parkinson's disease questionnaire 8. NEADL; Nottingham Extended Activities of Daily Living Test. UPDRS; unified Parkinson's disease rating scale. HAMD; Hamilton depression scale. PDQ39; Parkinson's disease questionnaire 39. WBDD-EO; weight bearing distribution difference – with eyes open. ML; Mediolateral. MBI; Modified Barthel index. BDI; Beck Depression Inventory. BESTest; Balance evaluation system test. LOS; limit of stability test. SLS; single leg stance test. FRT; functional reach test. HADS; hospital anxiety and depression. MFI; multidimensional fatigue inventory. GDS; geriatric depression scale. N/A; not available.
